# Prevalence of Injuries during Brazilian Jiu-Jitsu Training

**DOI:** 10.3390/sports5020039

**Published:** 2017-06-12

**Authors:** Alex R. McDonald, Fred A. Murdock, Josh A. McDonald, Christopher J. Wolf

**Affiliations:** 1 Department of Physical Medicine and Rehabilitation, University of Missouri, Columbia, MO 65212, USA; murdockf@health.missouri.edu (F.A.M.J.); wolfch@health.missouri.edu (C.J.W.); 2 Department of Biology, University of Wisconsin, Madison, WI 53706, USA; mcdonald4@wisc.edu

**Keywords:** Brazilian jiu-jitsu, martial arts, combat sports, training injury

## Abstract

Brazilian jiu-jitsu (BJJ) is a martial art that focuses on groundwork, joint locks, and chokeholds. The purpose of this study is to determine the prevalence of injuries sustained during BJJ training. A 27-question research survey was e-mailed to 166 BJJ gyms in the United States. Demographic information, belt level, weight class, training hours, competition experience, and injury prevalence data were collected. The majority of respondents were Caucasian (*n* = 96) males (*n* = 121) with an average age of 30.3 years. Overall, the most common injury locations were to the hand and fingers (*n* = 70), foot and toes (*n* = 52), and arm and elbow (*n* = 51). The most common medically diagnosed conditions were skin infections (*n* = 38), injuries to the knee (*n* =26), and foot and toes (*n* = 19). The most common non-medically diagnosed injuries occurred to the hand and fingers (*n* = 56), arm and elbow (*n* = 40), and foot and toes (*n* = 33). In general, athletes were more likely to sustain distal rather than proximal injuries. Athletes reported more frequent medically diagnosed injuries to the lower extremity and more frequent self-diagnosed injuries to the upper extremity. Upper extremity injuries appear to be more frequent but less severe than lower extremity injuries with the opposite being true for lower extremity injuries.

## 1. Introduction

Brazilian jiu-jitsu (BJJ) is a martial art that focuses on groundwork, joint locks, and chokeholds instead of kicks and punches. The sport has Japanese roots, and is specifically influenced by the technical aspects of Kodokan judo [1]. BJJ came into the spotlight when Royce Gracie won the 1st and 2nd Ultimate Fighting Championship (UFC) tournaments in the early 1990s [1,2]. Today, BJJ tournaments continue to increase in popularity [3]. The graduation system in BJJ rewards athletes with different colored belts to represent their progress in experience and knowledge. According to the International Brazilian Jiu-Jitsu Federation (IBJJF), the belt progression after the age of 17 is as follows: White, Blue, Purple, Brown, Black, Red and black, Red and white, and Red. Each belt has a minimum age requirement, and after receiving a blue belt, adults must hold each belt for a specified amount of time before progressing to the next. The maximum weight allowed in each weight class depends upon the athletes’ age and sex. The IBJJF weight classes for adults are as follows: Rooster (Male = 57.6 kg, Female = 48.5 kg), Light Feather (M = 64.2, F = 53.5), Feather (M = 70.1, F = 58.5), Light (M = 76.2, F = 64.2), Middle (M = 82.3, F = 69.2), Medium Heavy (M = 88.5, F = 74.2), Heavy (M = 94.3, F = 79.4), Super Heavy (M = 100.7, F = No maximum), Ultra Heavy (M = No maximum) [3].

Athletes within the same weight class and belt level compete against one another. During competition, matches are overseen by a referee who enforces the rules and regulations of the sport while simultaneously awarding points. Athletes can score points via takedowns, sweeps, and obtaining positional control over their opponents. Matches last 2–10 min depending on athlete age and belt level. These matches may end early via submission, loss of consciousness, athlete disqualification, referee stoppage, or random pick (if both athletes become injured). If no winner is determined before the match ends, victory is awarded by point score or referee decision [3]. 

Prior studies in similar grappling sports, such as wrestling and judo, have shown the impact of injuries to practitioners [4,5]. Despite an increased interest in BJJ, minimal research regarding the prevalence of injuries during competition has been conducted [6,7], and no research to our knowledge has evaluated the prevalence of injuries during training. The purpose of our study is to add to the available knowledge of injury prevalence in BJJ by investigating injuries that occur during training.

## 2. Materials and Methods

The Institutional Review Board at our academic center reviewed and approved this project. A 27-question research survey was e-mailed to 166 BJJ gyms publicly listed as members of the IBJJF and/or the Gracie Academy. Athletes over the age of 18 with or without injuries sustained in the last year were included in this study. Eight participants were excluded from the study for not meeting this inclusion criteria. Demographic information, belt level, weight class, training hours, competition experience, and injury prevalence data were collected. Athletes were asked to select injuries sustained within the prior year that occurred during BJJ specific training but not competition or conditioning. Respondents could indicate more than one injury but were asked to list each individual injury only once. Survey participants were incentivized by entrance into a random drawing to receive one of four $25.00 pre-paid credit cards.

## 3. Results

**Figure 1:**
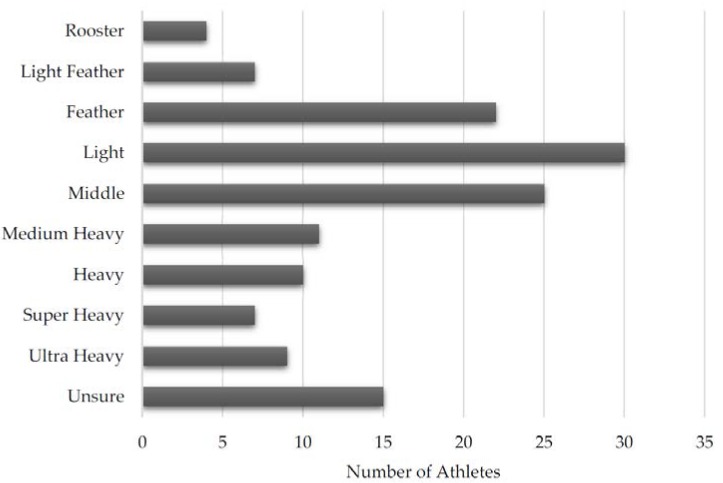
Distribution of athletes by weight class.

**Figure 2:**
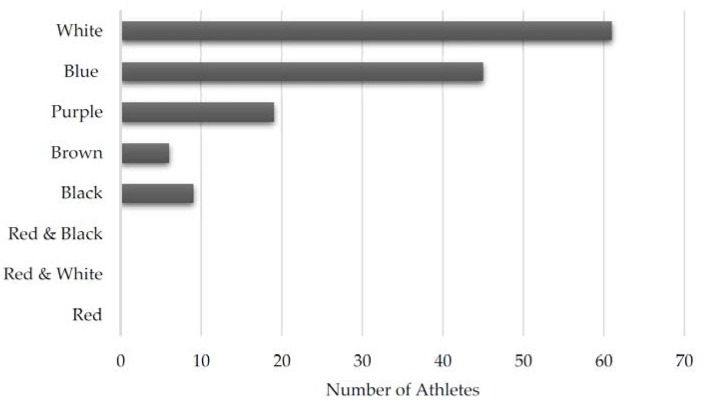
Distribution of athletes by belt level.

To analyze the training injuries of BJJ athletes, we reviewed questionnaire results from 140 survey participants who reported 487 total injuries; 120 of 140 (85.7%) participants reported an injury while 20 denied being injured. Complete athlete demographic information is shown in Table 1. The majority of the respondents were Caucasian (*n* = 96, 68.6%) males (*n* = 121, 87.1%) with an average age of 30.3 years and age range from 18 to 55 years. The distribution of athletes by weight class is shown in Figure 1. The two most common weight classes were Light (*n* = 30, 21.4%) and Middle (*n* = 25, 17.9%) weight. The distribution of athletes by belt level is found in Figure 2. Individuals with less experience were more likely to respond, with most athletes having White belts (*n* = 60, 42.2%) and Blue belts (*n* = 45, 32.4%). No respondents had obtained a Red and black, Red and white, or Red belt.

Athletes selected their level of participation in BJJ as one of the following: non-competitor who uses BJJ as a form of exercise (*n* = 36, 25.7%), non-competitor who trains for defense (*n* = 21, 15.0%), amateur competitor (*n* = 77, 55.0%), and professional competitor (*n* = 6, 4.3%). The average athlete participates in BJJ-specific training 3.7 days per week (StDev 1.56, median 4, range 7), totaling 7.63 h per week (StDev 4.87, median 6.25, range 39), and competes in 2.18 competitions per year (StDev 2.57, median 2, range 14). 

Injuries were differentiated as medically diagnosed or self-diagnosed. Medically diagnosed injuries are those evaluated by a medical professional. Self-diagnosed injuries are self-reported injuries the athlete did not seek medical attention for. Self-diagnosed injuries were only included in this study if they prevented the athlete from participating in BJJ training for at least one week. This was done to separate minor injuries from more clinically significant injuries affecting their participation in sport. 

**Figure 3:**
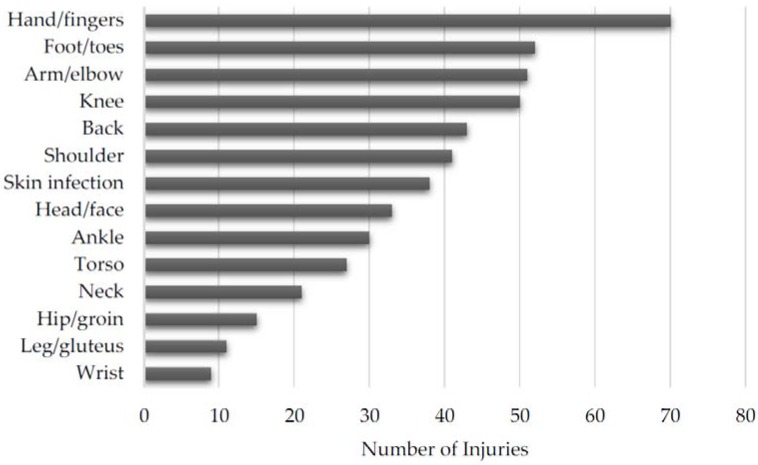
Overall distribution of injuries by anatomic location.

**Figure 4:**
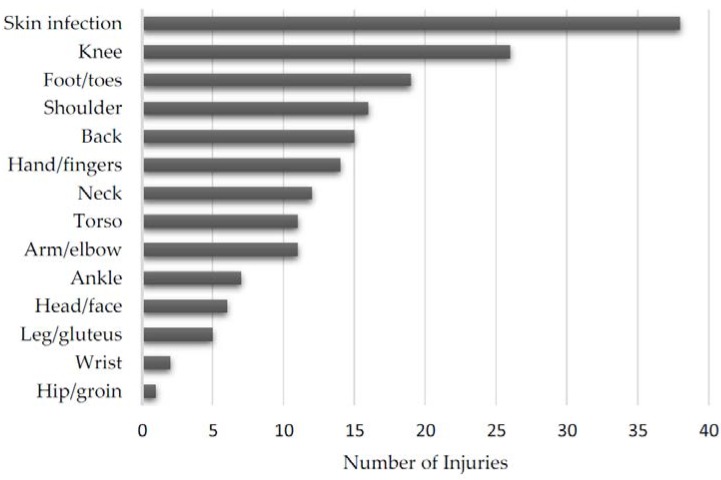
Distribution of medically diagnosed injuries by anatomic location.

**Figure 5:**
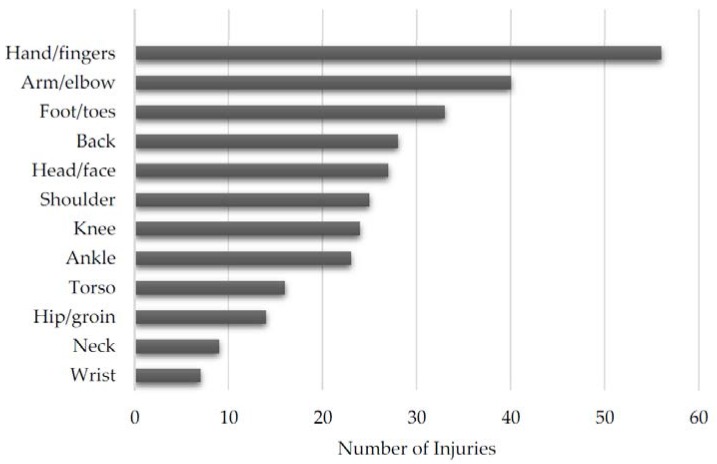
Distribution of self-diagnosed injuries by anatomic location.

Each individual reported injury can be found in Table 2. The overall distribution of injuries is shown in Figure 3 and the distribution of medically and self-diagnosed injuries can be found in Figure 4 and Figure 5, respectively. Additionally, the distribution of injuries by age, belt level, and weight class can be found in Table 3, Table 4 and Table 5, respectively.

## 4. Discussion

BJJ is an increasingly popular martial art but little is known about the incidence and prevalence of injuries in the sport [3]. Prior research has identified the incidence rate of injury in competition but has not addressed training injuries [6,7]. This is the first study to our knowledge that addresses the prevalence of injuries during BJJ training. Discovering the prevalence of injuries in training is important because athletes spend more time in training than in competition. As evidenced by our study, the average athlete participates in BJJ specific training approximately four days per week but only competes in two tournaments per year. Athletes, coaches, and medical professionals may use this information to better understand what injuries are more likely to occur in training.

Overall, the most common injuries occurred to the distal extremities. The two most common injuries occurred to the hands and fingers and the foot and toes. The third and fourth most injured locations were slightly more proximal and occurred to the arm and elbow and the knee. This pattern of increased distal injury potentially has a sport-specific etiology. The hands and fingers have much contact time with the opponent and are used to grasp the opponent’s clothing and body to maintain control, increasing the potential for injury. The feet and toes of the BJJ athlete may be at an increased risk of injury because athletes are barefoot when they train. Moreover, both fingers and toes can get caught in the opponent’s clothing or the gym mat the athletes train on.

Skin infections were the most common medically diagnosed injury. We hypothesize that athletes tend to seek medical treatment for skin infections more frequently than other injuries due to concern of spreading a potentially contagious infection to their training partners; other injuries would not directly affect other athletes. However, skin infections may simply be more prevalent. 

Our study indicates that athletes were more likely to seek medical attention for lower extremity injuries. Injuries to the knee and foot/toes were the second and third most common body parts to be injured. In contrast, the most common self-diagnosed injuries involved the upper extremities: hands/fingers, and arm/elbow. Injuries to the upper extremities occur more frequently in training but are not significant enough to warrant medical attention, while lower extremity injuries occur less frequently but are more severe. However, athletes may perceive lower extremity injuries as more significant causing them to seek medical care.

Scoggin et al. found elbow injuries to be the most prevalent orthopedic injury during BJJ competition [7]. Similarly, Kreiswirth et al. found elbow and knee injuries to have the same rate of injury in competition [6]. However, our study found that the hand/fingers followed by foot/toes were the most commonly injured body locations in training. The difference is likely due to the differing goals of training and competition. In competition, athletes can win a match via submission. Athletes apply extreme stress to their opponent’s joints to force them to “tap-out” and resign the match. This may result in injury especially if the opponent refuses to tap-out. In contrast, during training athletes are less likely to put equivalent stress on their opponent’s joints via submission to avoid injuring their training partner. 

Wrestling and judo are grappling sports similar to BJJ. Agel et al. found the most common injuries in collegiate wrestling practice to be skin infections (17.2%), knee injuries (14.8%), and ankle injuries (7.3%) [4]. This is comparable to our finding that skin infections and knee injuries are the most common medically diagnosed injuries. In the sport of Judo, Green et al. found finger and knee injuries to be the most prevalent injuries in competition; data on training injuries is not available[5]. This finding is consistent with our data that hand and finger injuries were the most common overall injuries in BJJ training.

The goal of this research is to help practitioners of BJJ prevent injury. Coaches can focus their education and develop their athletes with our results in mind. They can emphasize teaching students how to avoid the most common injuries with proper technique. Similarly, the athlete can better recognize the potential for injuries if a certain body part is being manipulated by an opponent. For example, the athlete can tap-out faster while training to avoid injury. Also, skin infections were the most common medically diagnosed condition; spread of such infections can be reduced if awareness is increased.

This study has potential limitations. We sent our survey to BJJ gym directors via publicly available e-mail addresses and asked them to forward our survey to their adult students. Therefore, we do not know how many athletes ultimately received our survey and are unable to calculate a response rate. Recall bias may have affected this study because respondents were asked to list injuries that occurred within the last year. Determining the etiology of each individual injury was beyond the scope of this study. A prospective study following a cohort of BJJ athletes would be helpful in determining both the etiology and incidence rate of injuries in practice. Despite the limitations of this study, we believe it contributes useful information on training injuries in BJJ athletes across the United States.

## 5. Conclusions

The goal of this epidemiological study is to determine the prevalence and most common injuries sustained during BJJ training. Respondents were allowed to report medically diagnosed and self-diagnosed injuries. Overall, the most common injuries occurred to the hands, fingers, feet, and toes. This finding indicates athletes were more likely to sustain distal rather than proximal injuries. Skin infections were the most common medically diagnosed condition, while the most common self-diagnosed injuries were to the hands and fingers. Athletes reported more frequent medically diagnosed injuries to the lower extremity and more frequent self-diagnosed injuries to the upper extremity. Upper extremity injuries appear to be more frequent but less severe when compared to lower extremity injuries in the BJJ athlete.

## Figures and Tables

**Table 1:** Athlete demographics.

Demographic	*n*	Percentage (%)
**Age**		
18–29	72	51.4
30–39	54	38.6
40–49	12	8.6
50–59	2	1.4
Sex		
Male	121	87.1
Female	19	12.9
**Race**		
American Indian or Alaska Native	0	0
Asian	14	10
Black or African American	3	2.1
Hispanic or Latino	12	8.6
Native Hawaiian or Other Pacific Islander	1	0.7
White	96	68.6
Other Race	2	1.4
Two or More Races	6	4.3
Prefer not to answer	6	4.3

**Table 2:** List of all injuries reported by athletes.

Medically Diagnosed Injury	Injuries (*n*)	Self-Diagnosed Injury	Injuries (*n*)
**Head and Face (*n* = 32, 6.6%)**			
Concussion	3	Black Eye	13
Eye injury	1	Cauliflower ear	11
Other	1	Broken tooth	1
		Other	2
**Neck (*n* = 21, 4.3%)**			
Muscle injury	5	Cervical pain	9
Disc injury	4		
Ligament/tendon strain	2		
Vertebral fracture	1		
Trachea injury	0		
**Back (*n* = 43, 8.8%)**			
Disc injury	5	Lower back pain	13
Muscle spasms	3	Muscle spasms	9
Other	3	Upper back pain	6
Muscle injury	2		
Vertebral fracture	1		
Spondylolysis/-listhesis	1		
**Torso (*n* = 27, 5.5%)**			
Fractured ribs	6	Chest pain	13
Other	4	Abdominal pain	3
Abdominal muscle injury	1		
Clavicle fracture	0		
**Shoulder (*n* = 41, 8.4%)**			
Rotator cuff injury	7	Shoulder pain	25
AC joint separation	3		
Dislocation	3		
Other	2		
Labrum tear	1		
**Elbow (*n* = 51, 10.5%)**			
Elbow hyperextension	4	Hyperextension	26
Arm/forearm muscle	2	Elbow pain	12
Strain/sprain	2	Arm/forearm pain	2
Elbow fracture	1		
Bone fracture	1		
Ligament/tendon tear	1		
Dislocation	0		
**Wrist (*n* = 9, 1.8%)**			
Bone fracture	1	Wrist pain	7
Sprain/strain	1		
**Hand and Finger (*n* = 70, 14.4%)**			
Jammed finger	4	Jammed finger	28
Other	4	Finger hyperextension	22
Bone fracture	3	Finger pain	6
Finger hyperextension	2		
Dislocated finger	1		
Ligament/tendon tear	0		
**Hip and Groin (*n* = 15, 3.1%)**			
Other	1	Hip pain	8
Bone fracture	0	Groin pain	6
Dislocation	0		
Ligament/tendon	0		
Muscle injury	0		
**Leg and Gluteal (*n* = 11, 2.3%)**			
Quad/hamstring injury	2	Leg pain	5
Bone fracture	1	Gluteal pain	1
Calf injury	1		
Gluteus injury	1		
**Knee (*n* = 45, 9.2%)**			
Meniscus tear	12	Knee pain	19
Ligament/tendon tear	7		
Knee sprain/strain	5		
Patella dislocation	1		
Other	1		
**Ankle (*n* = 30, 6.2%)**			
Sprain	4	Sprain	22
Bone fracture	2	Ankle pain	1
Ligament/tendon tear	1		
Achilles tendon	0		
**Foot and Toes (*n* = 52, 10.7%)**			
Bone fracture	7	Jammed toe	15
Turf toe	5	Toe hyperextension	13
Jammed toe	2	Foot or toe pain	5
Toe dislocation	2		
Lisfranc injury	1		
Toe hyperextension	1		
Other	1		
Ligament/tendon tear	0		
**Skin (*n* = 40, 8.2%)**			
Laceration requiring stitches	2		
Folliculitis/carbuncle/furuncle	2		
Impetigo	3		
Cellulitis	1		
Staph infection (unsure)	5		
Molluscum Contagiosum	3		
Verrucae	3		
Ringworm	15		
Other	6		

**Table 3:** Distribution of injuries sustained by age group.

Age	Injured Athletes (*n*)	Injured Athletes (%)	Most Common Injury (*n*)
18–29	62	51.7	Hand/fingers (26)
30–39	44	36.7	Hand/fingers (24)
40–49	12	10	Neck (6)
50–59	2	1.7	Skin infection (1), Foot/toes (1), Hand/fingers (1), Torso (1), Laceration (1)

**Table 4:** Distribution of injuries sustained by belt level.

Belt Level	Injured Athletes (*n*)	Injured Athletes (%)	Most Common Injury (*n*)
White	51	42.5	Hand/fingers (21)
Blue	39	32.5	Hand/fingers (18)
Purple	16	13.3	Knee (10), Hand/fingers (10)
Brown	6	5	Knee (4), Hand/fingers (4)
Black	8	6.7	Hip/groin (4)

**Table 5:** Distribution of Injuries Sustained by Weight Class.

Weight Class	Injured Athletes (*n*)	Injured Athletes (%)	Most Common Injury (*n*)
Rooster	4	3.3	Shoulder (4)
Light feather	6	5.0	Hand/fingers (5), Skin infection (5)
Feather	18	15.0	Hand/fingers (11)
Light	26	21.7	Knee (15)
Middle	22	18.3	Hand/fingers (10), Arm/elbow (10)
Medium heavy	9	7.5	Hand/fingers (5)
Heavy	8	6.7	Shoulder (4)
Super heavy	6	5.0	Hand/fingers (5)
Ultra heavy	8	6.7	Torso (4), Skin infection (4)
Unsure	13	10.8	Arm/elbow (5), Hand/fingers (5), Foot/toes (5)
